# Oxychloride of Zinc

**Published:** 1872-12

**Authors:** R. R. Freeman


### Oxychloride of Zinc.

BY R. R. FREEMAN.

*Read Before the Tennessee State Dental Society.*

This material is known under different names, as Osteoplasty, Os-Artificial Cement-Plombe, Guillois' Cement, etc., all having about the same properties, and used to accomplish the same end. It has no doubt attracted as much attention of late as is the case of anything presented for the consideration of our profession. It has some strong advocates, while there are those who seem strongly opposed to it. Some pronounce it a grand success, while others have failed entirely to bring about its accredited results.

All however who have used it to any considerable extent, admit that in certain cases there is nothing known to equal it. As a non-conductor it is extensively used; and careful operators invariably place it in the bottom of large cavities where they desire to fill with gold, and thermal changes are likely to cause irritation. The preparation known as os-artificial is probably best for this purpose, as it sets more readily than some of the others.

It is especially adapted where caries have been rapid, and there is such tenderness that you are unable to make proper retaining points. Prepare the cavity as thoroughly as possible, then apply the os-artificial, you will find it adherring close to the walls, the chloride of zinc relieves the tooth of its tenderness. After this filling has remained for a time, you may cut it out, make your retaining points. and fill permanently with but little inconvenience to the patient.

Many complaints are made on account of its washing out. And the question has been asked: Has any one succeeded in making a filling as hard as the samples we get on the boxes? I answer unhesitatingly, yes. You must not expect to arrive at good results without great care. He who will use it in a careless and indifferent manner had best discard it altogether. The hardness and durability of all osteoplastic fillings depend on absolute dryness: this can be best secured by using the rubber dam. Apply your dam with the same care you would in making a large adhesive gold filling; then insert your cement, being careful about inclosing air in the cavity; let it stand for half an hour, and finish up as de- desired after you have burnished as smooth as possible. Coat with wax, rubbing it on with a hot burnisher.

My own experience is not very extensive in the use of osteoplasty; it is sufficient however to make me a strong advocate of its claims.

I have record of a number of cases where for large molar fillings over softened dentine it has stood the test for two years, without an indication of change. For filling some temporary teeth I think it well adapted, even when the nerves are exposed you may relieve the pain, and retain the tooth in position until the permanent is ready to be irrupted. You should first relieve the tooth of all congestion; this is most readily done by bleeding it; then flood the cavity well with carbolic acid and iodine in the parts; dry out the cavity again carefully, place in the cement, let it harden, and depend upon it, in very few cases will you have any further trouble.

The introduction of osteoplasty has superceeded the use of the forceps to a considerable extent, particularly so in regard to six year molars. These teeth, from the age of ten to fourteen years, are frequently very defective. Decay is found to be so extensive, that if not alreadly exposing the pulp death is apt to follow from irritation, caused by thermal changes. We do not desire to destroy the pulps, treat and fill, for at best such teeth are only expected to last a few years, and when extracted produce deformity which is bound to follow through life. How much better, you would say, to extract such teeth before the fourteenth year molars were in place, then the space would fill up, and the bicuspids and anterior teeth be less crowded; and though this practice may tend, as has been said, to contract the jaws of our race we are satisfied that you had done the best thing under all the circumstances for the patient. Three good six year molars are frequently sacrificed on account of a defective one, in order, we say, to present an unbroken arch. If there be three defective and one good, the good one is invariably extracted.

How different with osteoplasty at hand; now we have command of the situation, and are able to save hundreds of teeth that have been heretofore sacrificed.

By the proper applications we may remove all inflammation, and even where pus is present healthy action may be again established. You may then cap with os-artificial; nature being assisted will at once commence her repairs, by a deposit of secondary dentine, and thus you may save tooth, pulp and all for permanent use.

Some make bold to deny that a pulp may be exposed, treated as above indicated, and yet retain its vitality; but those who have tested it know that it is not only possible but has actually been done time and time again.

I had a case where a bicuspid pulp was badly exposed, in full view, and bleeding from a wound. It was treated with carbolic acid and iodine, then filled with os-artificial, expecting after a short time, if no trouble followed, to fill permanently. The patient was called out of the State; did not see him again for a year, at the end of which time he called, with his tooth giving considerable pain. On examination the filling was found to be considerably washed, (not expecting to make a permanent filling, moisture was allowed to strike it before it had sufficiently hardened.) There was still a portion of the filling over the pulp, which on being removed disclosed the pulp still alive, and considerably congested.

